# Evaluation of cystatin C and microalbuminuria as predictive markers for hypertension-related renal complications

**DOI:** 10.1186/s41065-025-00557-7

**Published:** 2025-10-17

**Authors:** Jinyu Li, Shiming Guan, Zhuoqun Zou, Lijuan Yang, Bo Qian, Li Shen, Ye Li

**Affiliations:** Department of Geriatrics, Shanghai Health and Medical Center (Huadong Sanatorium), 67 Dajishan, Wuxi, 214065 Jiangsu Province China

**Keywords:** Combined laboratory test, Correlation to blood pressure, Cystatin c, Hypertension, Microalbuminuria

## Abstract

**Objective:**

This study aimed to investigate the correlation between blood pressure and the levels of two renal function markers, cystatin C and microalbuminuria, with the goal of evaluating their correlation across varying blood pressure categories.

**Methods:**

A cohort of 3,000 participants, comprising 1,500 individuals with a diagnosis of hypertension and 1,500 normotensive controls, was analyzed. Data on blood pressure, serum cystatin C, microalbuminuria, and other relevant clinical parameters were collected at admission. Correlations between these markers and blood pressure levels were analyzed using SPSS 26.0 statistical software.

**Results:**

Levels of cystatin C and microalbuminuria were significantly higher in individuals with hypertension compared to the control group (*P* < 0.05). Furthermore, both biomarkers demonstrated a positive correlation with blood pressure (*r* = 0.140, *P* < 0.001). Multiple linear regression analysis revealed that the combined use of cystatin C and microalbuminuria provided a superior predictive value for blood pressure levels (*P* < 0.001). The combined predictive accuracy (R² = 0.114. R² = 0.112 after rectifying age, body mass index, and blood lipid levels) exceeded that of cystatin C (R² = 0.100) or microalbuminuria (R² = 0.113) when analyzed individually.

**Conclusion:**

The biomarkers cystatin C and microalbuminuria are closely correlated with blood pressure levels. Their combined assessment offers an enhanced diagnostic approach for the early detection of renal complications in individuals with hypertension.

## Introduction

With the global increase in lifestyle-related changes and an aging population, the prevalence of hypertension is on the rise. Early detection and intervention in hypertension-related organ damage are crucial for preventing and managing hypertension and its associated complications. The kidneys are often among the first organs to be affected by hypertension, and a range of biomarkers has been employed to detect impairments in renal function. Among these, cystatin C and microalbuminuria have garnered significant attention in recent years as indicators of renal function. While prior studies have demonstrated a strong association between both cystatin C and microalbuminuria with hypertension-related renal impairment, there is limited research focusing on the combined use of these biomarkers in laboratory tests across different blood pressure categories [1, 2]. Furthermore, the correlation between simultaneous measurements of cystatin C and microalbuminuria and hypertension-induced renal impairment remains insufficiently explored. This study aims to investigate the correlation between blood pressure and the levels of cystatin C and microalbuminuria across varying blood pressure categories, with the goal of providing scientific evidence to support early diagnosis and risk assessment of hypertension-related renal complications.

## Materials and methods

### Study participants

A total of 3,000 participants were included in this study. They were divided into two groups: the hypertension group and the healthy control group, each consisting of 1,500 individuals. Based on the *2024 Hypertension Diagnostic Criteria*, the hypertension group was further categorized into three subgroups: grade 1 hypertension (*n* = 1,180), grade 2 hypertension (*n* = 204), and grade 3 hypertension (*n* = 116). The specific classification criteria are as follows: (1) Grade 1: Systolic blood pressure 140–159 mmHg and/or diastolic blood pressure 90–99 mmHg; (2) Grade 2: Systolic blood pressure 160–179 mmHg and/or diastolic blood pressure 100–109 mmHg; (3) Grade 3: Systolic blood pressure ≥ 180 mmHg and/or diastolic blood pressure ≥ 110 mmHg. Participants in the hypertension group were individuals undergoing initial physical examinations at the Shanghai Health and Medical Center and met the following inclusion criteria: (1) Age between 35 and 65 years. (2) Systolic blood pressure ≥ 140 mmHg and/or diastolic blood pressure ≥ 90 mmHg. (3) No history of diabetes mellitus, chronic renal disease, or other conditions (e.g. use of diuretics) known to affect blood pressure or renal function. The healthy control group comprised individuals undergoing routine physical examinations at the same health checkup center during the same period. These individuals were matched to the hypertension groups by age and gender and had no history of hypertension, cardiovascular diseases, or diabetes mellitus.

Participants consumed a normal diet of approximately 1.5 g/kg/day of protein and did not engage in regular exercise.

### Detection methods

Blood pressure measurements of the study participants were obtained using an approved OMRON electronic sphygmomanometer. After a minimum rest period of 20 min in a quiet environment, blood pressure was measured at the brachial artery of the upper arm in the seated position. Three consecutive measurements were taken at two-minute intervals, and the average value of these readings was recorded as the blood pressure. Venous blood and morning urine samples were collected in a fasting state from all participants. Serum cystatin C levels were measured using enzyme-linked immunosorbent assay (ELISA) methods. Blood lipid and glucose levels were measured using an automatic biochemical analyzer. Microalbuminuria (urine albumin) was measured in 24-hour urine collections using immunoturbidimetry using an automatic urine analyzer, which detected antigen–antibody complex formation by measuring absorbance at 340 nm to calculate albumin concentration. All procedures were conducted in strict adherence to the manufacturer’s instructions for the respective assay kits.

### Statistical analysis

Data were analyzed using SPSS 26.0 statistical software. Normality of continuous variables was assessed using the Shapiro–Wilk test. An independent-samples t-test was employed to compare differences between the hypertension and the healthy control groups. Additionally, one-way analysis of variance (ANOVA) was used to assess variations within and between the groups. Spearman’s correlation test was conducted to evaluate the relationship between blood pressure levels and the levels of cystatin C and microalbuminuria, as blood pressure was treated as an ordinal variable by hypertension grade and the biomarker–blood pressure relationship may not be strictly linear. The predictive accuracy of combined cystatin C and microalbuminuria laboratory tests in predicting blood pressure-related complications was further assessed through multiple linear regression analysis.

## Results

### Analysis of cystatin C levels

The mean cystatin C levels in the hypertension group were 0.87 ± 0.16 mg/L, compared to 0.82 ± 0.14 mg/L in the healthy control group. Independent-samples t-test results indicated that cystatin C levels were significantly higher in the hypertension groups compared to the healthy control group (t value: -7.690, *P* < 0.001) (Table 1). One-way analysis of variance (ANOVA) further revealed significant differences in cystatin C levels among the healthy group and hypertension groups (F value: 20.369, *P* < 0.001) (Table 2). Cystatin C level exhibited a gradual increase with rising blood pressure (Table 2).

**Table 1:** Comparison of cystatin C and microalbuminuria levels between healthy control and hypertension groups ($\:\stackrel{-}{\text{x}}\:$± s)

Group	Healthy Control (*n* = 1500)	Hypertension (*n* = 1500)	t-value	*P*-value
Cystatin C (mg/L)	0.82 ± 0.14	0.87 ± 0.16	-7.690	< 0.001
Microalbuminuria (mg/g)	21.2 ± 14.5	28.1 ± 19.6	-8.571	< 0.001

**Table 2:** Comparison of cystatin C and microalbuminuria levels between healthy controls and different hypertension subgroups ($\:\stackrel{-}{\text{x}}\:$± s)

Group	Healthy Control (*n* = 1500)	Grade 1 Hypertension (*n* = 1180)	Grade 2 Hypertension (*n* = 204)	Grade 3 Hypertension (*n* = 116)	F-value	*P*-value
Cystatin C (mg/L)	0.82 ± 0.14	0.87 ± 0.15	0.89 ± 0.12	0.93 ± 0.24	20.369	< 0.001
Microalbuminuria (mg/g)	21.2 ± 14.5	27.7 ± 19.4	31.0 ± 21.2	32.3 ± 20.9	25.775	< 0.001

### Analysis of microalbuminuria

Independent-samples t-test results showed that microalbuminuria levels were significantly elevated in the hypertension groups compared to the healthy control group (t value: -8.571, *P* < 0.001) (Table 1). Specifically, the average microalbuminuria level in the hypertension groups was 28.1 ± 19.6 mg/g, while the healthy control group had an average level of 21.2 ± 14.5 mg/g. One-way analysis of variance (ANOVA) showed significant differences in microalbuminuria level among the groups (F value: 25.775, *P* < 0.001) (Table 2). These findings support the use of microalbuminuria as an important indicator of renal function impairment in individuals diagnosed with hypertension.

### Analysis of correlation between cystatin C and microalbuminuria

Pearson’s correlation analysis demonstrated a statistically significant but modest positive correlation between cystatin C and microalbuminuria (*r* = 0.140, *P* < 0.001), indicating that elevated cystatin C levels are correlated with higher microalbuminuria levels. These findings suggest that the two biomarkers may exert a synergistic effect in reflecting renal impairment related to elevated blood pressure.

### Regression analysis of blood pressure, cystatin C, and microalbuminuria

Multiple linear regression analysis indicated that the combined assessment of cystatin C and microalbuminuria effectively predicted blood pressure levels (*P* < 0.001). The predictive accuracy of the combined model (R² = 0.114, adjusted to R² = 0.112 after controlling for age, body mass index, and blood lipids) was higher compared to models using either cystatin C (R² = 0.100) or microalbuminuria (R² = 0.113) individually. These results highlight the importance of combining both biomarkers for improved diagnosis and risk assessment of hypertension-related renal complications.

## Discussion

### Role of cystatin C and microalbuminuria in blood pressure-related diseases

Cystatin C and microalbuminuria have emerged as significant biomarkers in recent years, particularly in their ability to reflect changes in renal function [3–6]. Hypertension is a major contributor to renal dysfunction, with early indicators often involving a reduction in glomerular filtration rate (GFR) and compromised glomerular barrier function [7]. Cystatin C serves as a sensitive marker of GFR, with elevated levels signifying a decline in renal filtration efficiency [8]. Similarly, microalbuminuria indicates damage to the glomerular filtration barrier. The combined assessment of cystatin C and microalbuminuria provides a more comprehensive evaluation of renal function impairments [9]. In hypertension, elevated blood pressure increases glomerular capillary pressure, leading to endothelial dysfunction and subtle injury to the glomerular filtration barrier, which ultimately resulting in microalbuminuria [10]. Renal structural changes and reduced glomerular filtration can elevate serum cystatin C levels, reflecting early impairment of kidney function even before overt creatinine changes are detectable [11]. Cystatin C was measured as part of routine blood tests, and urinary microalbumin was routinely assessed to evaluate target organ damage in patients with hypertension [12].

The findings of this study indicate that individuals with hypertension exhibit significantly higher levels of cystatin C and microalbuminuria compared to the healthy control group. Furthermore, a positive correlation between cystatin C and microalbuminuria was observed, suggesting that elevated blood pressure levels are correlated with greater renal impairment in patients with hypertension. The combination of these biomarkers provides a more precise assessment of such impairments, consistent with previous research findings. This reinforces the clinical relevance of cystatin C and microalbuminuria as early indicators of hypertension-induced renal dysfunction [13, 14]. 

### Correlation between blood pressure levels and combined laboratory tests of cystatin C and microalbuminuria

The results of this study further demonstrate that cystatin C and microalbuminuria levels are correlated with blood pressure levels. Additionally, laboratory assessments that combine these biomarkers significantly elevate the accuracy of predicting blood pressure-related diseases. These results hold considerable clinical significance, as the combined use of cystatin C and microalbuminuria facilitates earlier and more accurate identification of potential renal function impairments among individuals with hypertension [15]. Additionally, the use of these biomarkers offers a valuable approach to evaluating the future risk of more severe cardiovascular complications in individuals with hypertension [16].

Most recent studies on cystatin C and microalbuminuria have focused on the diagnostic and prognostic value of either biomarker individually in chronic renal disease and diabetic nephropathy [17, 18]. In contrast, this study is among the first to systematically analyze the correlation between blood pressure and the combined use of cystatin C and microalbuminuria, particularly emphasizing their combined value in hypertension. The results of the multiple regression analysis demonstrate that the combination of cystatin C and microalbuminuria provides significantly greater accuracy in predicting blood pressure levels compared to either biomarker alone. This suggests the potential utility of these biomarkers in tandem as an important approach for assessing hypertension-related renal dysfunction.

### Clinical applications and future research directions

The findings of this study indicate that laboratory assessments incorporating both cystatin C and microalbuminuria provide robust support for the early diagnosis and risk assessment of hypertension-related renal impairments. The combined use of these biomarkers facilitate earlier detection of renal dysfunction in patients with hypertension and assist clinicians in developing individualized treatment plans, ultimately reducing the risk of severe cardiovascular events.

Future research should explore the clinical utility of combining cystatin C and microalbuminuria in diverse hypertension populations, including those with primary and secondary hypertension. In addition, as a cross-sectional study, this study was unable to capture the dynamic changes in cystatin C and microproteinuria levels over time. Longitudinal studies will be valuable in elucidating the roles of these biomarkers in the progression of hypertension, particularly in understanding the temporal relationship between their levels and the onset of hypertension-related complications. Long-term follow-up studies may also yield a more precise understanding of the mechanisms involved.

Although the sample size in this study was sufficient to achieve statistical significance, certain limitations exist, particularly regarding the regional characteristics and population selection, which may affect the generalizability of the results. Therefore, future large-scale, multicenter studies will be required to verify the applicability of combined cystatin C and microalbuminuria testing across broader populations.

In summary, laboratory tests combining cystatin C and microalbuminuria hold significant promise in the early diagnosis and risk assessment of hypertension-related renal function impairments. Future studies should focus on further evaluating their potential across diverse hypertension populations and investigating their role in guiding personalized treatment and prognosis assessment.

## Conclusion

This study evaluated and analyzed cystatin C and microalbuminuria levels in individuals with hypertension. The findings revealed that both cystatin C and microalbuminuria levels are significantly elevated in the hypertension groups compared to the healthy control group. Additionally, a positive correlation was observed between the levels of these biomarkers and blood pressure. These results suggest that laboratory assessments combining cystatin C and microalbuminuria serve as a significant reference for the early diagnosis and risk assessment of hypertension-related renal function impairments.

## Data Availability

The datasets used and/or analysed during the current study available from the corresponding author on reasonable request.

## Declarations

Clinical trial number: not applicable.

## References

[1] Jiang FM, Zhu XL, Zu HY. Diagnostic value of joint determination of urine microalbumin and serum cystatin C levels in patients with hypertension early renal injury. China Mod Doctor. 2018;74(5):157–8.

[2] Liu R. Clinical significance of detection of urinary microalbumin in early renal damage in elderly patients with hypertension. Guide China Med. 2020;18(26):91–2.

[3] Wang HF, Dong C, Li CM. Clinical value of combined detection of serum β 2-microglobulin, urinary microalbumin, and urinary N-acetyl - β - glucosidase in the diagnosis of early renal injury in hypertension. Shaanxi Med J. 2020;49(7):897–9.

[4] Cai GQ, Gou J, Jiao LT. Biological characteristics and clinical application evaluation of cystatin C. Int J Lab Med. 2006;27(5):457–60.

[5] Fox CS, Matsushita K, Woodward M, et al. Associations of kidney disease measures with mortality and end-stage renal disease in individuals with and without hypertension: a meta-analysis. Lancet. 2023;380:1649–61. 10.1016/S0140-6736(12)61272-0 PMC3993095 23013600

[6] Colombo M, Looker HC, Farran, B, et al. Serum kidney injury molecule 1 and β2-microglobulin perform as well as larger biomarker panels for prediction of rapid decline in renal function in type 2 diabetes. Diabetologia. 2019;62(1):156–68. 30288572 10.1007/s00125-018-4741-9 PMC6290680

[7] Shlipak MG, Katz R, Kestenbaum B, et al. Clinical and subclinical cardiovascular disease and kidney function decline in the elderly. Atherosclerosis. 2023;231:528–33. 10.1016/j.atherosclerosis.2008.08.016 PMC2696894 18848325

[8] Benoit SW, Ciccia EA, Devarajan P. Cystatin C as a biomarker of chronic kidney disease: latest developments. Expert Rev Mol Diagn. 2020;20(10):1019–26. 10.1080/14737159.2020.1768849 32450046 10.1080/14737159.2020.1768849 PMC7657956

[9] Lees JS, Welsh CE, Celis-Morales CA, et al. Glomerular filtration rate by differing measures, albuminuria and prediction of cardiovascular disease, mortality and end-stage kidney disease. Nat Med. 2019;25(11):1753–60. 10.1038/s41591-019-0627-8 31700174 10.1038/s41591-019-0627-8 PMC6858876

[10] Drożdż D, Drożdż M, Wójcik M. Endothelial dysfunction as a factor leading to arterial hypertension. Pediatr Nephrol. 2023;38(9):2973–85. 10.1007/s00467-022-05802-z 36409370 10.1007/s00467-022-05802-z PMC10432334

[11] Khader NA, Kamath VG, Kamath SU, Rao IR, Prabhu AR. Kidney function estimation equations: a narrative review. Ir J Med Sci. 2025;194(2):725–43. 10.1007/s11845-025-03874-y 39873963 10.1007/s11845-025-03874-y PMC12031902

[12] Wu S, Li S, Huang J, et al. The association between blood pressure variability and renal damage in patients with primary aldosteronism. J Clin Hypertens (Greenwich). 2024;26(7):765–71. 10.1111/jch.14824 38689511 10.1111/jch.14824 PMC11232444

[13] Stevens LA, Schmid CH, Greene T, et al. Factors other than glomerular filtration rate affect serum cystatin C levels. Kidney Int. 2023;72:495–504. 10.1038/ki.2008.638 PMC4557800 19119287

[14] Freedman BI, Iskandar SS, Appel RG. The link between hypertension and ephrosclerosis. Am J Kidney Dis. 2023;25:207–21. 10.1016/0272-6386(95)90001-2 7847347

[15] Zhang L, Sun J, Zhang M, et al. The significance of combined detection of cysc, urinary mAlb and β2-MG in diagnosis of the early renal injury in pregnancy-induced hypertension syndrome. Saudi J Biol Sci. 2019;26(8):1982–5. 10.1016/j.sjbs.2019.07.013 31889782 10.1016/j.sjbs.2019.07.013 PMC6923450

[16] Alzaabi MA, Abdelsalam A, Alhammadi M, et al. Evaluating biomarkers as tools for early detection and prognosis of heart failure: a comprehensive review. Card Fail Rev. 2024;10:e06. 10.15420/cfr.2023.24. Published 2024 Jun 3. 38915376 10.15420/cfr.2023.24 PMC11194781

[17] Taal MW, Brenner BM. Renal risk scores: progress and prospects. Kidney Int. 2024;64(2):377–8. 10.1038/ki.2008.36 18322541

[18] Niu Q, Gao R. Clinical value analysis of urinary microalbumin detection in evaluating elderly hypertension and early renal damage (Chinese). J Front Med. 2021;11(18):58–9.

